# The role of artificial intelligence in Latin American ruminant production systems

**DOI:** 10.1093/af/vfae034

**Published:** 2025-01-04

**Authors:** Einar Vargas-Bello-Pérez, Oscar R Espinoza-Sandoval, Manuel Gonzalez Ronquillo, Juan Carlos Angeles Hernandez, Alfonso J Chay-Canul, Héctor Aarón Lee-Rangel, Germán Danilo Amaya, Juan Pablo Keim, Javier Baudracco, Ricardo Augusto Mendonça Vieira, Navid Ghavipanje

**Affiliations:** Facultad de Zootecnia y Ecología, Universidad Autónoma de Chihuahua, Chihuahua, México; Department of International Development, School of Agriculture, Policy and Development, University of Reading, Reading, UK; Facultad de Zootecnia y Ecología, Universidad Autónoma de Chihuahua, Chihuahua, México; Facultad de Medicina Veterinaria y Zootecnia, Departamento de Nutrición Animal, Universidad Autónoma del Estado de México, Toluca, México; Departamento de Medicina y Zootenia de Rumiantes, Facultad de Medicina Veterinaria y Zootecnia, Universidad Nacional Autónoma de México, Mexico City, CP. 04510, Mexico; División Académica de Ciencias Agropecuarias, Universidad Juárez Autónoma de Tabasco, Villahermosa, Tabasco, México; Facultad de Agronomía y Veterinaria, Universidad Autónoma de San Luis Potosí, San Luis Potosí, Mexico; Department of School of Agriculture, Policy and Development, University of Reading, Reading, UK; Faculty of Agricultural Sciences, Animal Production Institute, Universidad Austral de Chile, Valdivia, Chile; IciAgro Litoral, Universidad Nacional del Litoral-CONICET, Kreder 2805 Esperanza (3080), Argentina; Laboratório de Zootecnia, Centro de Ciências e Tecnologias Agropecuárias (CCTA), Universidade Estadual do Norte Fluminense Darcy Ribeiro (UENF), Campos dos Goytacazes, RJ, Brazil; Faculty of Agriculture, Department of Animal Science, University of Birjand, Birjand, Iran

**Keywords:** artificial intelligence, machine learning, new technologies, ruminants, sustainability

**Implications**

- Artificial intelligence (AI) offers various technologies that can enhance both the quantity and quality of ruminant production in Latin America through precision management, data-driven decision-making, early disease detection, and environmental monitoring.
- Challenges of using AI in Latin American ruminant production include high costs, complexity, data requirements, risk of technological displacement, ethical and social implications, and regulatory and legal frameworks.
- Further research is needed to fully understand the potential impact of AI on the ruminant sector, including evaluating economic benefits, ethical considerations, and long-term effects on the industry and surrounding communities.

## Introduction

Today, cutting-edge technologies—internet of things (IoT), big data, cloud computing, and artificial intelligence (AI)—play a forefront role in sustainable livestock production (Morrone et al., 2022; Dayoub et al., 2024; Melak et al., 2024). AI, a subfield of computer science that simulates human intelligence enables real-time monitoring and analytical processing in the ruminant sector to boost production efficiency and animal welfare (Neethirajan, 2023; Dayoub et al., 2024).

With over 360 million head of cattle, Latin America is a key driver in global ruminant production and represents a remarkable agricultural frontier over the past 50 years (Figueroa et al., 2022). While Latin America makes up only 16% of the global population, it accounts for 23% of beef cattle and 24% of dairy cattle production worldwide, generating 30% of the planet’s meat and 28% of its bovine milk (FAO, 2021). Hence, the ruminant sector in this region drives 46% of the agricultural gross domestic product (GDP; Figueroa et al., 2022). For long-term sustainable planning of the ruminant sector in Latin America, aligning with global trends is essential.

AI is already applied in Latin American agriculture for precision farming, predictive analytics, supply chain optimization, resource management, and market forecasting (Vitón et al., 2019; Silvi et al. 2021; Puntel et al., 2022). Although compared to the leading countries (the United States and China) that have adopted AI in agriculture, this region is lagging in the development of smart technologies, and there is a need for academia to respond to the existing gaps together with the industry. Below we summarized AI impacts on Latin American ruminant systems, informing priority-setting in upcoming research initiatives and policy frameworks promoting AI adoption.

## Literature Research Methodology

The study took place in April 2024. We reviewed scientific articles in electronic databases including Web of Science, PubMed, CAB Abstracts, and Google Scholar from 2000 to 2024 using the following keywords “artificial intelligence,” “Latin America,” “ruminants,” “machine learning,” and “digital agriculture.” Then a critical evaluation of the most relevant paper abstracts resulted in 230 scientific reports. Those that best fit and were most relevant to the review topic were selected. To complete this process, important viewpoints, trends, and results that were relevant to the research objectives were identified.

## Latin American Leading Countries on the Use of AI in Animal Science

**Figure 1.**
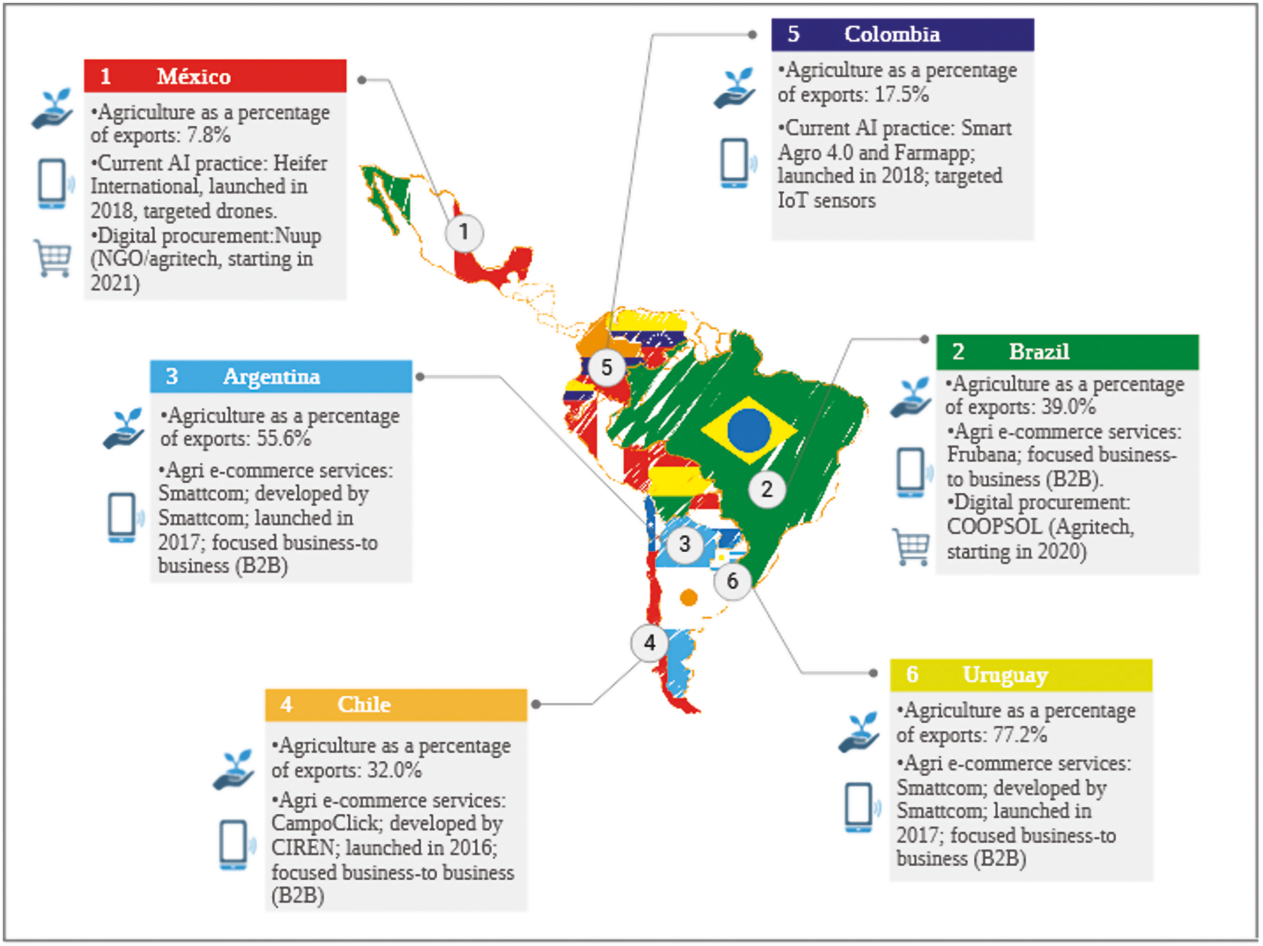
Agriculture in Latin America: current state and adoption of AI. Abbreviations: NGO, nongovernmental organization; IoT, internet of things; CIREN, natural resource information center.

The following section will discuss realities from some Latin American countries that have been using AI tools in animal science (Figure 1). However, before discussing the specifics, it is important to note some context on what these countries represent to this geographical region. Here are a few of the most developed countries in Latin America, ordered by their nominal GDP in 2024:

### Brazil

The first economy in Latin America the eighth in the world, and the only member of the BRICS in Latin America, hosts a multifaceted, resource-rich economy supported by agricultural terrain, ores, and the Amazon rainforest (Azzoni, 2001). Global prominence ensues in agriculture, specifically soybean, sugarcane, coffee, and cattle markets, thanks to innovations and expanded access (Swart and Brinkmann, 2020). Brazil’s economic growth raises ecological concerns like Amazon deforestation, impacting ecosystems, and climatic change, which prioritizes green growth for future development (Swart and Brinkmann, 2020).

### Mexico

The second economy in Latin America, and the 13th in the world, made significant headway in economic growth and metamorphosis. Mexico attained macroeconomic stability with low inflation, fiscal discipline, and stable currency supported by prudent monetary, and fiscal policies, and structural reforms (Furtado, 2018). Mexico has a highly open economy deeply intertwined with the United States through trade agreements (Nash, 2021). Mexico continues confronting challenges like poverty, inequity, and security necessitating efforts in comprehensive expansion and institutional modernization (Nathaniel et al., 2021).

### Argentina

Transitioning from 19th-century agricultural prominence to 20th-century economic instability (Della Paolera and Taylor, 2003), still heavily reliant on agriculture—primarily soybeans, corn, and wheat—fluctuates global commodity costs and sales (Diamand, 2019). Ranks fourth and sixth worldwide in beef and dairy exporters, respectively (considering the European Union as a single supplier), Argentina harbors potentials, such as a skilled workforce, rich natural reserves, and a multifaceted economy. However, rectifying structural issues and nurturing sustainable growth remains paramount for Argentina’s forward momentum (Regúnaga and Rodriguez, 2015).

### Chile

Stands out as a Latin America leader with its market-driven economy that is rooted in liberalized trading practices, effective macroeconomic administration, and commitment to free enterprise (Panez et al., 2020). Its diversified economy spans mining (particularly copper), agriculture, manufacturing, and technology mainly developed on trade relations through trade agreements (Hernandez and Madeira, 2022). Fueling innovation, entrepreneurship, and technological progress make it ascend as a regional digital hotspot; however, ensuring inclusive growth remains crucial for sustaining Chile’s long-term economic success (Madeira, 2022).

### Colombia

Experienced substantial economic expansion and change over past decades, featuring diverse industries like agriculture, mining, manufacturing, services, and energy (Schneider and Hametner, 2014). Boosted by abundant natural riches—namely oil, coal, gold, and arable terrain—the Colombian economy grew, garnered ample exports, and demonstrated recession resiliency (Vargas-Carpintero et al., 2023). Overall, Colombia’s economic development has been characterized by resilience, diversification, and improvements in living standards. Continued efforts to address social challenges, promote inclusive growth, and strengthen institutions will be essential for sustaining Colombia’s economic progress in the long term.

### Uruguay

Uruguay offers a high living standard, strong social welfare, and political stability (Furtado, 2018). It has a diversified economy including agriculture, services, manufacturing, and tourism, and reduced dependence on a single industry (Hofman and Valderrama, 2021). Uruguay ranks highly as a key player in exporting beef, soybeans, rice, and dairy goods while preserving macroeconomic balance via responsible financial management, reduced inflation, and steady currency rates (Awosusi et al., 2022). Future success hinges on sustained initiatives promoting sustainability, tackling societal hurdles, and enhancing institutional frameworks.

## Application and Advantages of AI in Ruminant Production in Latin America

**Figure 2.**
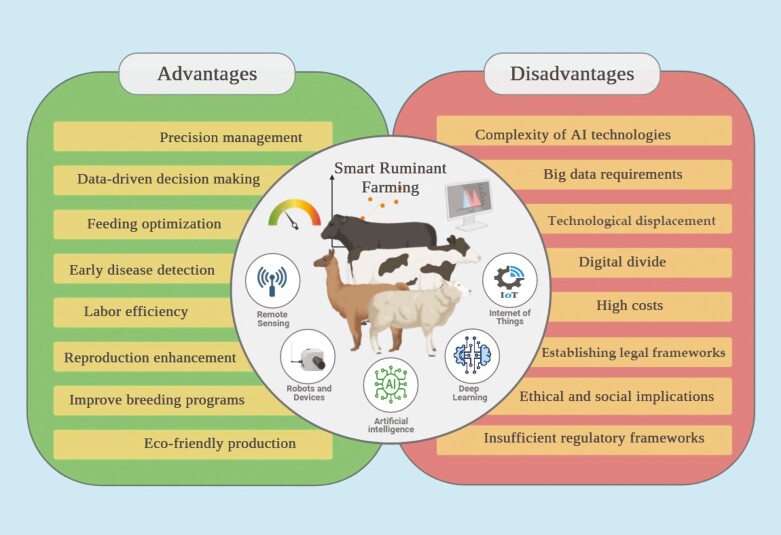
Advantages and disadvantages of using AI in Latin American ruminant production.

The AI revolution is creating rapid changes in all types of ruminant farming and plenty of research and developments on AI are booming, partly due to hopes and claims regarding its possible advantages for ruminant production (Curti et al., 2023; Neethirajan, 2023; Melak et al., 2024). In Latin America, current scientific reports and applications of AI in ruminants cover a range of topics aimed at improving productivity, welfare, and sustainability in livestock production that offer several advantages (Figure 2). Here are some areas where AI is being applied:

### Feeding and nutrition management

Globally, research trends indicated that data arising from AI models (such as data fusion and ML techniques) can be combined in different ways to achieve objectives such as optimizing feed intake, feed efficiency, nutrients retained or excreted, and reducing feed costs (Marzetti, 2022; Melak et al., 2024). AI algorithms can also develop personalized feeding programs tailored to each animal’s nutritional needs, growth stage, and health status (Melak et al., 2024). To keep up with global trends, in Latin America, da Rosa Righi et al. (2020) designed an innovative model targeting dairy cattle management optimization in Brazilian agricultural settings using IoT devices. In this model, MooCare performs the automation and individualization of the animal’s feeding complemented by accurate daily milk production forecasts per cow, realized through the implementation of the auto-regressive integrated moving average-based predictive algorithm. Besides, the notification system was able to forecast nutritional issues, allowing the farmers to design individualized plans for specific cows. Vásquez et al. (2019) designed an expert system integrating fuzzy logic modeling to gauge the effects of varying factors on milk and meat yield in México, aiming to facilitate informed choices for sustainable agrosystems. Smallholder dual-purpose cattle farming dominates Latin America. Oriana et al. (2021) examined 383 rural operations for reproductive technologies and found low levels of technology adoption among farmers. Application and execution of automated milking machines are documented in Argentina (Millapán et al., 2023), México (Negrete, 2020), and Chile (Melendez et al., 2019), assisted by AI technologies, enabling close observation of behaviors, health, nutrition, and reproductive cycles for prompt issue identification, and maximized oversight. These promising reports underscore that leveraging AI-driven models, and data derived from disparate sources including but not limited to sensors, visual recordings, and digital platforms can offer advantages for ruminant feeding and nutrition management in Latin America including precision management, optimized feeding, reducing feed costs, reducing waste, and minimizing environmental impact.

### Reproduction, diseases, and health management

Arteaga-Troncoso et al. (2023) predicted the abortion burden of several microorganisms in sheep in México using both an artificial neural network (ANN) and the generalized linear model showing the favorable performance of these models. Similarly, Freitas et al. (2023) evaluated the feasibility of machine learning (ML) methods using phenotypic traits to predict sheep gastrointestinal nematodes in Brazil that ultimately benefit farm-level decision-making. de Sousa et al. (2018) introduced an ANN model forecasting individual rectal temperatures in Brazilian feedlot steers, useful for gauging heat stress severity, whereas Teixeira et al. (2022) confirmed recurrent neural networks prowess in spotting incipient anaplasmosis outbreaks in young female Brahman-cross calves utilizing rumination and movement data gathered by HR Tag sensors.

In Latin America, AI technologies can assist in reproductive management, also, smart technology and cutting-edge software make it simpler than ever to keep an eye on the living conditions of the animals and spot any anomalies that might harm them. Also, AI could be applied for predicting estrus cycles, optimizing breeding schedules, and monitoring fertility parameters. These improve reproductive efficiency, increase conception rates, and enhance genetic selection outcomes, leading to improved herd productivity and profitability. AI-based systems can monitor physiological and behavioral data—vital signs, movement patterns, and feeding behavior—to detect early signs of disease in ruminants. Pedometers and other systems are used to detect lameness in both dairy and beef cattle. Sensor’s analysis has also been used for ML and achieved high precision in identifying lameness three days before visible symptoms. Additionally, AI shows potential in detecting calving illnesses like mastitis and ketosis in dairy cows’ mammary glands. In short, the advantages of AI in this sector in Latin America include enhanced reproduction management, predicting estrus cycles, monitoring fertility parameters, facilitating selective breeding programs, safeguarding animal health, and early disease detection.

### Environmental monitoring and sustainability

AI-based systems analyze farm environment—temperature, humidity, pasture quality, and water—via sensors and satellite data, aiding farmers make informed managerial decisions. Veintimilla et al. (2022) developed a system for monitoring and geolocation of cattle using the IoT. The system was developed using a Pycom device, which can integrate WiFi, Figfox, Bluetooth, FiPy, and LTE. Pybytes was the used web platform where the temperature and GPS position data were received and integrated into webhooks, and then the user was notified using a messenger program. In Latin American ruminant systems, AI technologies bring great opportunities to analyze environmental data to assess factors such as temperature, humidity, and pasture quality, helping farmers optimize resource management and minimize environmental impact. This promotes sustainable agricultural practices, reduces greenhouse gas emissions, and enhances ecosystem health and resilience (INIA, 2021; García et al., 2024).

### Predictive modeling and decision support systems

AI-driven predictive models offer precision forecasts of key ruminants like milk yield and growth rates helping farmers optimize production and mitigate risks. AI tools analyze large volumes of data from various sources, including sensors, satellite imagery, and historical records, to provide actionable insights for farm management (Neethirajan, 2023; Melak et al., 2024). In Latin America, Gomez-Vazquez et al. (2024) developed an advanced ML technique, supported by principal component analysis, to estimate body weight in buffaloes in México highlighting the potential of this ML approach in sustainable animal production decision-making. Marín-Urías et al. (2023) created a prediction model to improve the Bull Breeding Soundness via ANNs, this approach enhanced decision-making by 12% compared to traditional breeding bull management. Asthana (2014) demonstrated that ML models can efficiently optimize cattle ranches operations offering easy implementation, enhanced profitability, and effective incentive strategies for managers. The acceptable performance of ML techniques to identify the ideal weight of cattle in Colombia was also approved by García et al. (2021). Of note, the National Agricultural Research Institute (INIA) Uruguay introduced the INIA Thermostress—a decisional framework centered on mitigating risks of climate incidents—that provides exact temperature and humidity index predictions. This instrument also empowers dairy and livestock producers to deploy timely counteractions against extreme heat in animals (INIA, 2021). In Colombia, Garcia et al. (2024) devised an independent surveillance apparatus for observing beef cattle fattening procedures, incorporating anomaly discovery and explanation modules. Their invention reduces abnormalities and displays remarkable diagnostic accuracy, revealing the potential of infusing self-regulating competencies into production platforms. Collectively, these works underscore AI’s untapped potential to assist farmers, allowing them to reduce resource usage, enhance the sustainability of their feeding practices, optimize farm productivity, reduce labor costs, and support managerial decision-making in Latin American ruminant systems.

## Challenges of Using AI in Ruminant Production in Latin America

While AI offers numerous benefits for ruminant production in Latin America, there are also several potential disadvantages and challenges associated with its use:

### Digital infrastructure and divide

The absence of digital infrastructure poses a critical challenge for Latin America in supporting its AI ecosystem (Patel et al., 2021). It has been reported that multinational companies are attempting to establish regional hubs in the region, yet the essential infrastructure, such as 4G, 5G, and fiber optics, remains insufficient. Mexico, Chile, and recently Brazil are the only countries in Latin America with existing 5G infrastructure (Patel et al., 2021). The issue is aggravated by the digital divide, and the disparities in access to and use of information and communication technologies between different groups of people. In rural areas of Latin America, farmers may lack access to AI technologies and digital tools, exacerbating existing inequalities in agricultural development. Additionally, the lack of cooperation between researchers, engineers, and technology suppliers leads to the low adoption rate of AI technologies in Latin America (Galvez and Galvez, 2024). These disparities could widen the gap between tech-savvy farmers and their less digitally inclined counterparts, potentially reinforcing patterns of marginalization within the agricultural community.

### Cost

The adoption of AI in the ruminant sector involves costs and is complicated by ever-changing AI architecture, driven by continuous innovation and development (Morrone et al., 2022). In Latin America, rising costs for small-scale farming operations have become increasingly burdensome and smaller farms might find it difficult to adopt AI technology due to its high cost (Mont et al., 2020; Galvez and Galvez, 2024). A survey conducted by Bolfe et al. (2020) targeting 753 rural producers, companies, and service providers in Brazil identified that 67% of these farmers and 58% of service providers consider investing in machinery, equipment, or applications as the principal challenge in implementing and maintaining digital transformation in agricultural operations. Therefore, it is essential to invest in the development of AI solutions tailored for use in small-scale ruminant production systems to address this concern effectively.

### Complexity

AI systems often require specialized knowledge and expertise to operate effectively. Farmers and technicians may need training and support to understand how to use AI tools, interpret results, and troubleshoot technical issues (Patel et al., 2021). Insufficient education and experience in AI may prevent ruminant farmers in Latin America from effectively using and optimizing AI tools while managing large volumes of AI-generated data can be problematic without proper professional support (Sanchez-Pi et al., 2021; Figueroa et al., 2022). Latin America is recognized as a minor contributor to global AI research. The region contributes merely 2.7% to peer-reviewed AI publications, trailing behind Sub-Saharan Africa with 0.7%, and is significantly outpaced by East Asia and the Pacific (36.9%), Europe and Central Asia (25.1%), North America (17.1%), and the MENA region (5.5%; Clark et al., 2021). It has been also reported that the integration of AI-related skills in the labor market is lower in Latin America (2.16%) than in the rest of the world (3.59%; Vera Ramírez, 2024). Therefore, the complexity of AI technology can be a barrier to adoption, especially in rural areas with limited access to training and technical assistance.

### Dependency on data and external inputs

Massive dataset requirement is identified as a major challenge accompanying AI applications in Latin America (Parrado, 2023). Most studies are focused on sensor technologies and analytical techniques; however, other steps are often overlooked. One example is the obstacles encountered during data collection and transfer in remote regions, such as larger areas that characterize the majority of beef cattle system production in Latin America (Curti et al., 2023). Addressing these gaps is imperative for effective AI implementation in Latin American extensive production systems. In addition, unreliable infrastructure or limited access to external inputs (i.e., electricity, internet connectivity, and maintenance services) in rural areas of Latin America can hinder AI leverage (Patel et al., 2021). Overreliance on AI technology could make farms more vulnerable to disruptions caused by equipment malfunctions or other external factors beyond human control and expose farms to increased risks and vulnerabilities, undermining long-term sustainability.

### Risk of innovation and technological displacement

The adoption of AI in ruminant production may lead to the displacement of traditional farming practices and manual labor. While AI can improve efficiency and productivity, it may also result in job losses or changes in employment patterns, particularly for workers engaged in routine tasks that can be automated. Thus, the risk of technological displacement poses yet another challenge for resource-strapped farmers seeking to remain competitive within evolving markets. Notably, despite widespread recognition of AI’s potential, its application’s assured success remains uncertain (D’Amour et al., 2022). In Latin America, factors such as steep initial costs and unpredictability contribute to underutilization, restricting numerous prospective benefits. In 2020, a mere 58% of companies in the region invested in AI (Sanchez-Pi et al., 2021).

### Ethical and social implications

AI leverage in all industries raises ethical issues like job displacement, data ownership concerns, and fair access to technology (Patel et al., 2021). In Latin America, where a substantial segment of the population works in agriculture (with 125 million people employed in the industry, accounting for 16% of the economically active population), automation and subsequent reductions in employment could result in grave implications, given the high unemployment rate (Galvez and Galvez, 2024). Moreover, animal welfare, genetic modification, and environmental footprint raise moral questions pertinent to AI’s presence in ruminant manufacture. Concentrating solely on productivity instead of animal health and ethics might ignite controversy regarding farming intensity, genetics, species diversity, and longevity.

## Regulatory and Legal Frameworks

Implementing AI in ruminant farming requires solid regulations securing user privileges, stimulating innovation, and establishing confidence among parties (Ordoñez et al., 2024). Swift AI advances bring legal complications tied to IP, data possession, culpability, and customer defense. Lawmakers must establish fitting rules for liability, moral AI adoption, weighing innovation, and precautions. At present, most countries in Latin America lack coherent legislation governing the development, deployment, and governance of AI systems, leaving farmers exposed to potential abuses and exploitation. Strengthening institutional capacities and policy interventions becomes crucial for navigating this emerging landscape successfully (Marzetti, 2022). Policymakers in Latin America should deliberate on the strategies to overcome these challenges and determine the appropriate policies to implement in the short and long term (Patel et al., 2021). It has been suggested that regulatory frameworks should implement strategies that attract researchers from leading countries and institutions to the Latin America region, establish AI programs in partnership with industry, and create public awareness while creating an AI research agenda focused on local and regional AI challenges (Patel et al., 2021; Español et al., 2023).

## Conclusion

In Latin America, the rapid progress of AI-powered technologies benefits ruminant farming and promotes eco-friendly and sustainable production, however, there are still obstacles to overcome. Issues such as high costs, big dataset requirements, risk of inaccurate predictions, ethical concerns, risk of technological displacement, and complexity, among others. Addressing these multifaceted challenges calls for collaborative efforts involving animal scientists, veterinarians, computer scientists, animal behavior specialists, and agri-engineers.

## About the Authors

 **Einar Vargas-Bello-Pérez** is a Doctor of Veterinary Medicine at Universidad Nacional Autónoma de México, and he holds MSc (McGill University, Canada) and PhD (University of Nottingham, United Kingdom) degrees. Currently, he is an Associate Professor at Universidad Autónoma de Chihuahua in México and a Visiting Research Fellow at the University of Reading in the United Kingdom. He has been an Associate Professor for Pontificia Universidad Católica de Chile, the University of Copenhagen, and the University of Reading. His research line is on the interface between ruminant nutrition and product quality. He is also devoted to animal production with special emphasis on the use of new technologies in livestock production systems that assure sustainability and welfare in the context of One-Health, One-Welfare, and One-Feed. His international research network includes institutions from Asia, the Middle East, North America, Europe, and Latin America. **Corresponding author:**einar.vargasbelloperez@reading.ac.uk; evargasb@uach.mx

 **Oscar R. Espinoza-Sandoval** grew up in a family dedicated to forage and dairy farming in the north of México, which is part of the second-largest dairy farmers association in the country, where he actively takes part in extension activities. In the past has worked in several areas of dairy science, including reproduction, nutrition, heat stress, and dairy farm simulation. Now his PhD research at the Universidad Autónoma de Chihuahua is focused on the development and integration of Artificial Intelligence models into decision support systems, to evaluate different strategies of management to improve the dairy farms’ decision process.

 **Manuel González Ronquillo** is a Specialist in Veterinary Medicine and Zootechnics, working in the Department of Animal Nutrition of the Faculty of Veterinary Medicine and Zootechnics of the Autonomous University of the State of Mexico. He obtained his PhD in Veterinary Medicine from the University of Zaragoza, Spain. He has worked at USDF research, Madison, WI; FAO-AGA division, Italy; and Universidad de Magallanes, Chile. His main research interests focus on sustainable animal diets and the effects of animal diets on the quality of food products of animal origin. In recent years, he has focused his work on diets for farm animals, with special attention to the reduction of methane emission in ruminants, the nonuse of antibiotics, and the use of indigenous resources of the region to reduce the carbon footprint.

 **Juan Carlos Ángeles Hernandez** is a researcher at the Department of Ruminant Medicine and Zootechnics, Faculty of Veterinary Medicine and Zootechnics, National Autonomous University of Mexico (UNAM). He graduated from UNAM with a PhD in Health Sciences and Animal Production. He has experience in the mathematical modeling of sheep lactation curves and in the calculation of uncertainty in greenhouse gas emission inventories using Monte Carlo and bootstrap simulation. Recent incursion into the application of machine learning tools in animal production using artificial intelligence.

 **Alfonso J. Chay Canul** holds a degree in Agricultural Engineering. He obtained his Master of Science in Tropical Animal Production with an option in Animal Nutrition and his Doctorate in Agricultural Sciences from the Autonomous University of Yucatan. Since February 2012, he has been a research professor in the Academic Department of Agricultural Sciences of the Universidad Juárez Autónoma de Tabasco. He is currently a member of the National System of Researchers Level 2. His research areas are ruminant nutrition, nutritional requirements of small ruminants, energy efficiency in ruminants, evaluation of local feed resources for ruminant feeding, and simulation models applied to animal production.

 **Héctor Aarón Lee Rangel** is a full-time professor at the Faculty of Agronomy and Veterinary Medicine at UASLP. He holds a bachelor’s degree in animal science and completed his master’s and doctoral studies focusing on ruminant nutrition. His research focuses on increasing propionate production and reducing ruminal methane through nonconventional additives and herbal phytogenics. His work includes basic research in metabolomics, nutrigenomics, and epigenetics. He has also investigated the mechanisms of feed intake regulation, a scientifically significant and productive area. Currently, he is exploring the application of machine learning in animal science.

 **Germán Danilo Amaya** is a PhD Candidate in International and Rural Development at the University of Reading. His research focuses on understanding the livelihoods of dairy farmers in the Colombian Andes, considering their assets and capabilities to adapt to climate change and market dynamics.

 **Juan Pablo Keim** is an Associate Professor at the Institute of Animal Production, Faculty of Agricultural and Food Sciences, Universidad Austral de Chile (UACh). He obtained his bachelor’s degree and PhD from Universidad Austral de Chile. His research interests are the evaluation of the ruminal fermentation of dairy cattle feed (in vitro, in situ, and in vivo methods), the use of forage resources (both pastures and supplementary crops) by dairy cows, relationships between environmental pollution and nutrition, and factors that affect the productivity of dairy systems. He has led and collaborated in projects financed by VIDCA-UACh, Fondecyt, and FIA, among others. He is responsible for teaching the subjects of Bovine Milk Production and Animal Feeding at the undergraduate level, and Ruminant Nutrition and Ruminant Nutrition Topics, among others where he collaborates.

 **Javier Baudracco** is a Professor of Dairy Production at the Universidad Nacional del Litoral and a researcher at the Institute IciAgro CONICET-UNL in Argentina. He holds a PhD in Animal Science and a Master of Science with First Class Honours from Massey University in New Zealand. Dr. Baudracco has served as the Director of Postgraduate Studies in Dairy Systems at his university and as the Director of Dairy Production for the National Government of Argentina. His research focuses on various aspects of dairy systems, including grazing, stocking rate, modeling, genetics, animal welfare, environmental impact, and profitability. With extensive practical experience, he worked as a dairy farm milking assistant and farm advisor. Dr. Baudracco is passionate about making dairy farming a sustainable and enjoyable business.

 **Ricardo Augusto Mendonça Vieira** is an animal scientist who graduated in 1993 and received his DSc in 1998, in Ruminant Nutrition. He has worked in quantitative ruminant nutrition since 1997 and has taught quantitative methods since 2000. As a full professor, Ricardo teaches Goat Production and Animal Feed Analysis to the undergrad students of Animal Science and Quantitative Aspects of Ruminant Nutrition for the Grad students in the Animal Science Graduate Program. Ricardo has advised more than 50 MS and DS students and published more than one hundred indexed papers in WoS or Scopus. His research interests rely on fiber, intake, efficiency of nutrient utilization, and general nonlinear phenomena in Animal Science.

 **Navid Ghavipanje** earned his PhD degree in Animal Nutrition from the Department of Animal Science of the University of Birjand, Iran. Navid centered his research on the impact of alternative ingredients, sustainable diets, and plant-based bioactive compounds on animal production, favoring the circular economy, reducing feed-food-fuel competition, and mitigating enteric methane emissions. He also contributed to the development of potentially healthier animal-derived foods for human nutrition. More recently, His scholarly journey was marked by the integration of artificial intelligence in animal nutrition and production, especially ruminants.
